# Analysing installation scenarios of Debian packages

**DOI:** 10.1007/978-3-030-45237-7_14

**Published:** 2014-12-15

**Authors:** Benedikt Becker, Nicolas Jeannerod, Claude Marché, Yann Régis-Gianas, Mihaela Sighireanu, Ralf Treinen

**Affiliations:** 8 Johannes Kepler University, Linz, Austria; 9 University of Birmingham, Birmingham, UK; 10 Université Paris-Saclay, Univ. Paris-Sud, CNRS, Inria, LRI, 91405 Orsay, France; 11 Université de Paris, IRIF, CNRS, 75013 Paris, France; 12 Inria, 75013 Paris, France

**Keywords:** Quality Assurance, Safety Properties, Debian, Software Package Installation, Shell Scripts, High-Level View of File Hierarchies, Symbolic Execution, Feature Tree Constraints

## Abstract

The Debian distribution includes more than 28 thousand maintainer scripts, almost all of them are written in Posix shell. These scripts are executed with root privileges at installation, update, and removal of a package, which make them critical for system maintenance. While Debian policy provides guidance for package maintainers producing the scripts, few tools exist to check the compliance of a script to it. We report on the application of a formal verification approach based on symbolic execution to find violations of some non-trivial properties required by Debian policy in maintainer scripts. We present our methodology and give an overview of our toolchain. We obtained promising results: our toolchain is effective in analysing a large set of Debian maintainer scripts and it pointed out over 150 policy violations that lead to reports (more than half already fixed) on the Debian Bug Tracking system.

## 1 Introduction

**The Debian distribution** is one of the oldest free software distributions, providing today 60 000 binary packages built from more than 31 000 software source packages with an official support for nine different CPU architectures. It is one of the most used GNU/Linux distributions, and serves as the basis for some derived distributions like Ubuntu.

A software package of Debian contains an archive of files to be placed on the target machine when installing the package. The package may come with a number of so-called *maintainer scripts* which are executed when installing, upgrading, or removing the package. A current version[4] of the Debian distribution contains 28 814 maintainer scripts in 12 592 different packages, 9 771 of which

* This work has been partially supported by the ANR project CoLiS, contract number ANR-15-CE25-0001.

[4] *sid* for *amd64*, including *contrib* and *non-free*, as of October 6, 2019

are completely or partially written by hand. These scripts are used for tasks like cleaning up, configuration, and repairing mistakes introduced in older versions of the distribution. Since they may have to perform any action on the target machine, the scripts are almost exclusively written in some general-purpose scripting language that allows for invoking any Unix command.

The whole installation process is orchestrated by `dpkg`, a Debian-specific tool, which executes the maintainer scripts of each package according to *scenarios*. The `dpkg` tool and the scripts require *root* privileges. For this reason, the failure of one of these scripts may lead to effects ranging from mildly annoying (like spurious warnings) to catastrophic (removal of files belonging to unrelated packages, as already reported [39]). When an execution error of a maintainer script is detected, the `dpkg` tool attempts an *error unwind*, but the success of this operation depends again on the correct behaviour of maintainer scripts. There is no general mechanism to simply undo the unwanted effects of a failed installation attempt, short of using a file system implementation providing for snapshots.

The *Debian policy* [4] aims to normalise, in natural language, important technical aspects of packages. Concerning the maintainer scripts we are interested in, it states that the standard shell interpreter is Posix shell, with the consequence that 99% of all maintainer scripts are written in this language. The policy also sets down the control flow of the different stages of the package installation process, including attempts of error recovery, defines how `dpkg` invokes maintainer scripts, and states some requirements on the execution behaviour of scripts. One of these requirements is the *idempotency* of scripts. Most of these properties are until today checked on a very basic syntactic level (using tools like *lintian* [1]), by automated testing (like the *piuparts* suite [2]), or simply left until someone stumbles upon a bug and reports it to Debian.

**The goal of our study** is to improve the quality of the installation of software packages in the Debian distribution using a formal and automated approach. We focus on bug finding for three reasons. Firstly, a real Unix-like operating system is obviously too complex to be described completely and accurately by some formal model. Besides, the formal correctness properties may be difficult to apprehend by Debian maintainers especially when they are expressed on an abstract model. Finally, when a bug is detected, even on a system abstraction, one can try to reproduce it on a real system and, if confirmed, report it to the authors. This has a real and immediate impact on the quality of the software and helps to promote the usage of formal methods to a community that often is rather sceptical towards methods and tools coming from academic research.

The bugs in Debian maintainer scripts that we attempt to find may come at different levels: simple syntax errors (which may go unnoticed due to the unsafe design of the Posix shell language), non-compliance with the requirements of the Debian policy, usage of unofficial or undocumented features, or failure of a script in a situation where it is supposed to succeed.

**The challenges** are multiple: The Posix shell language is highly dynamic and recalcitrant to static analysis, both on a syntactic and semantic level. A Unix file system implementation contains many features that are difficult to model, e.g., ownership, permissions, timestamps, symbolic links, and multiple hard links to regular files. There is an immense variety of Unix commands that may be invoked from scripts, all of which have to be modelled in order to be treated by our tools. To address properties of scripts required by the Debian policy, we need to capture the transformation done by the script on a file system hierarchy. For this, we need some kind of logic that is expressive enough, and still allows for automated reasoning methods. A particular challenge is checking the idempotency property for script execution because it requires relational reasoning. For this, we encode the semantics of a script as a logic formula specifying the relation between the input and the output of the script, and we check that it is equivalent to its composition with itself. Finally, all these challenges have to be met at the scale of tens of thousands of scripts.

**The contributions** of this work for this case study are:

1. A translation of Debian maintainer scripts into a language with formal semantics, and a formalisation of properties required for the execution of these scripts by the Debian policy.
2. A verification toolchain for maintainer scripts based on an existing symbolic execution engine [5,6] and a symbolic representation [26]. Some components of this toolchain have been published independently; we improve them to cope with this case study. The toolchain is free software available online [35].
3. A formal specification of the transformations done by an important set of POSIX commands [24] in *feature tree constraints* [26].
4. A number of bugs found by our method in recent versions of Debian packages.

We start in the next section with an overview of our method illustrated on a concrete example. Section 3 explains in greater detail the elements of our toolchain, the particular challenges, the hypotheses that we could make for the specific Debian use case at hand, and the solution that we have found. Section 4 presents the results we have found so far on the Debian packages, and the lessons learnt. We conclude in Section 5 by discussing additional outcomes of this study, the related and future work.

## 2 Overview of the case study and analysis methodology

### 2.1 Debian packages

Three components of a Debian binary package play an important role in the installation process: the *static content*, i.e., the archive of files to be placed on the target machine when installing the package; the lists of *dependencies* and *predependencies*, which tell us which packages can be assumed present at different moments; and the *maintainer scripts*, i.e., a possibly empty subset of four scripts called `preinst`, `postinst`, `prerm`, and `postrm`. We found (Section 4.2) that 99% of the maintainer scripts in Debian are written in POSIX shell [22].

Our running example is the binary package `rancid-cgi` [31]. It comes with only two maintainer scripts: `preinst` and `postinst`. The `preinst` script is included in Fig. 1. If the symbolic link `/etc/rancid/lg.conf` exists then it is

```
if [ -h /etc/rancid/lg.conf ]; then
  rm /etc/rancid/lg.conf
fi
if [ -e /etc/rancid/apache.conf ]; then
  rm /etc/rancid/apache.conf
fi
```

**Fig. 1.** `preinst` script of the `rancid-cgi` package removed; if the file `/etc/rancid/apache.conf` exists, no matter its type, it is also removed. Both removal operations use the POSIX command `rm` which, without options, cannot remove directories. Hence, if `/etc/rancid/apache.conf` is a directory, this script fails while trying to remove it.

We did a statistical analysis of maintainer scripts in Debian to help us design our intermediate language, see Section 4.2 for details. We found that, for instance, most variables in these scripts can be expanded statically and hence are used like constants; most `while` loops can be translated into `for` loops; recursive functions are not used at all; redirections are almost always used to discard the standard output of commands.

## 2.2 Managing package installation

The maintainer scripts are invoked by the `dpkg` utility when installing, removing or upgrading packages. Roughly speaking, for installation `dpkg` calls the `preinst` before the package static content is unpacked, and calls the `postinst` afterwards. For deinstallation, it calls the `prerm` before the static content is removed and calls the `postrm` afterwards. The precise sequence of script invocations and the actual script parameters are defined by informal flowcharts in the Debian policy [4, Appendix 9]. Fig. 2 shows the flowchart for the package installation. `dpkg` may be asked to: install a package that was not previously installed (Fig. 2), install a package that was previously removed but not purged, upgrade a package, remove a package, purge a package previously removed, remove and purge a package. These tasks include 39 possible execution paths, 4 of them presented in Fig. 2.

The Debian policy contains [4, Chapters 6 and 10] several requirements on maintainer scripts. This case study targets checking the requirements regarding the execution of scripts, and considers out of scope some other kinds of requirements, e.g., the permissions of script files. The requirements of interest are checked by different tools of our toolchain presented in Section 3. For example, the different ways to invoke a maintainer script are handled by the analysis of scenarios (Section 3.5) calling the scripts. Different requirements on the usage of the shell language are checked by the syntactic analysis (Section 3.1), like the usage of `-e` mode or of authorised shell features that are optional in the POSIX standard. Some of the usage requirements can be detected by a semantic analysis; this is done in our toolchain by a translation into a formally defined language, called CoLiS (Section 3.1). Finally, requirements concerning the be-

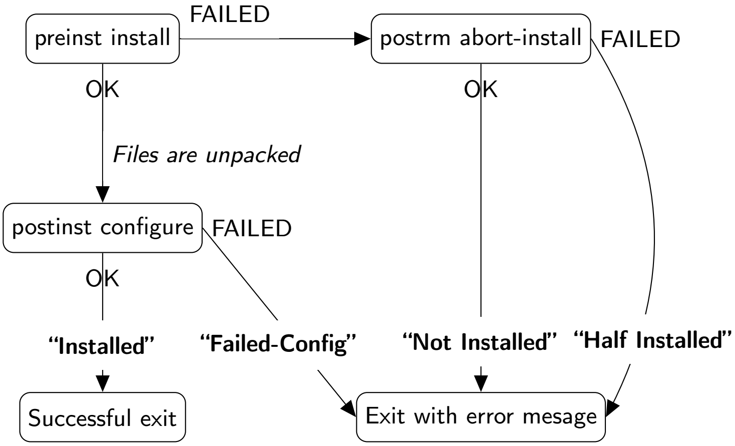

**Fig. 2.** Debian flowchart for installing a package [4, Appendix 9] (The states represent calls to maintainer scripts with their arguments and the status returned by `dpkg` at the end of the process is in bold.)

haviour of scripts include the usage of exit codes and the *idempotency* of scripts. The last property is difficult to formalise since it refers to possible unforeseen failures (see discussion in Section 4.4). Checking behavioural properties requires to reason about their semantics, which is done by a symbolic execution in our toolchain (Section 3.4). We also check some requirements that are simply common sense and that are not stated in the policy, e.g., invoking Unix commands with correct options. This is done by the semantic analysis (Section 3.1).

### 2.3 Principles and workflow of the analysis method

Our goal is to check the above properties of maintainer scripts in a formal way, by analysing each script and the composition of scripts in the execution paths exhibited by the flowcharts of `dpkg`. We call *scenario* either an execution path of `dpkg`, a single execution of a script, or a double execution of a script with the same parameters (to check idempotency); refer to Section 3.5 for more details.

The analysis should consider a variety of states for the system on which the execution takes place. Yet we assume the following hypotheses: the scripts are executed in a *root* process without concurrency with other user or *root* processes, the static content of the package is successfully unpacked, the dependencies defined by the package are present (fact checked by `dpkg`), and the `/bin/sh` command implements the standard Posix.1-2017 Shell Command Language with the additional features described in the Debian policy [4, Chapter 10].

The components of our toolchain for the analysis of a scenario are summarised on Fig. 3 and detailed in Section 3. Given a package and one scenario, the scenario player extracts the static content and the maintainer scripts, prepares the initial *symbolic state* of the scenario, symbolically executes the steps of the scenario to

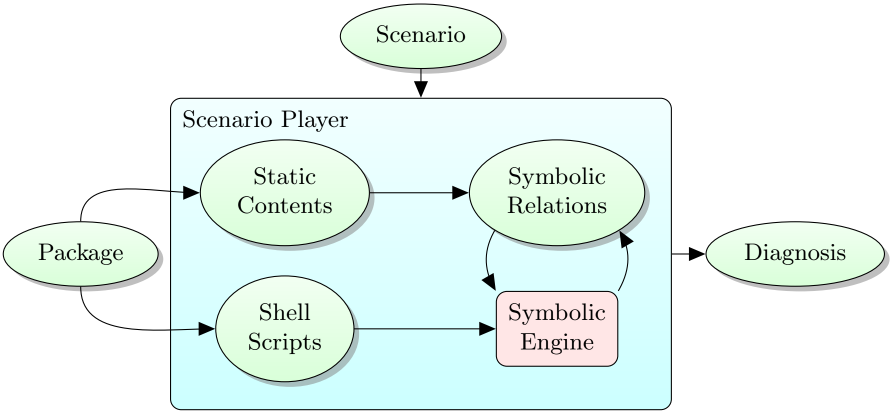

**Fig. 3.** Toolchain for analysis of a scenario on a given package (see Section 2.3)

compute a *symbolic relation* between the input and the output states of the file system for each outcome of the scenario, and produces a diagnosis.

### 2.4 Presentation of results

The results computed by the scenario player are presented in a set of web pages, one per scenario, and a summary page for the package [34]. Each scenario may have several computed exit codes; for an error code, the associated symbolic relation is translated automatically into a diagnosis message.

For example, consider the simple scenario of a call to the script `preinst` given in Fig. 1. The result web page includes the diagram in Fig. 4, which is obtained by the interpretation of the symbolic relation computed by the scenario player for the error exit code. The diagram represents an abstraction of the initial file system on the left, an abstraction of the file system at the end of the script's execution on the right, and the relation between these abstractions (dotted lines). In this diagram, a plain edge represents the parent relation in the file hierarchy. A dotted edge describes a similarity relation, e.g., the trees rooted at `/etc` coincide except on the child named `rancid`. ⊥ denotes the absence of a node. Finally, a leaf can be annotated by a property, e.g., the annotation `dir` rooted at `/etc/rancid/apache.conf`. The diagram shows that the `preinst` script leads to an error state when the file `/etc/rancid/apache.conf` is a directory since the `rm` command cannot remove directories.

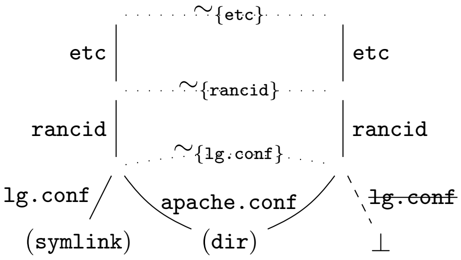

**Fig. 4.** Example of diagnosis: error case for `preinst` call in the package `rancid-cgi`

Finally, another set of generated web pages provides statistics on the coverage and the errors found for the full set of scenarios of the Debian distribution.

## 3 Design and implementation of the tool chain

The toolchain, as described in Fig. 3, hinges on a *symbolic execution engine* which computes the overall effect of a script on the file system as a symbolic relation between the input and the output file system. This section details this execution engine, which is composed of (i) a front-end that parses the script and translates it into a script in a formally defined intermediate language called CoLiS, and (ii) a back-end that symbolically executes the CoLiS scripts to get, for each outcome of the script, the relation between input and output file systems encoded by a *tree constraint*.

### 3.1 Front-end

*Shell parser.* The syntax of the Posix shell language is unconventional in many aspects. For this reason, the implementation of a parser for Posix shell cannot simply reuse the standard techniques solely based on code generators. Most of the shell implementations falls back to manually written character-level parsers, which are difficult to maintain and to trust. morbig [30] is a parser that tries to use code generators as much as possible to keep the parser implementation at a high level of abstraction, simplifying maintenance and improving our ability to check if it complies with the Posix standard.

*The CoLiS language.* It was first presented in 2017 [23]. Its design aims to avoid some pitfalls of the shell, and to make explicit the dangerous constructions we cannot eliminate. It has a clear syntax and a *formally* defined semantics. We provide an automated and direct translation from Posix shell. The correctness of the translation from shell to CoLiS cannot be proven formally but must be trusted based on manual review of translations and tests.

For this case study, we improved the language proposed formerly [23] to increase the number of analysed Debian maintainer scripts. First, we added a number of constructs to the language. Second, we provide a formal semantics for the new constructs and we align the previous semantics [23] to the one of the Posix shell for a few other constructs. These changes and a complete description of the current CoLiS language are described in a technical report [6]. Fig. 5 shows the CoLiS version of the `preinst` script of the `rancid-cgi` package, shown previously in Fig. 1. Notice the syntax for string arguments and for lists of arguments that requires mandatory usage of delimiters. Generally speaking, the syntax of CoLiS is designed so as to remove potential ambiguities [6].

The toolchain for analysing CoLiS scripts is designed with formal verification in mind: the syntax, semantics, and interpreters of CoLiS are implemented using the Why3 environment [7] for formal verification. More precisely, the syntax of CoLiS is defined abstractly (as abstract syntax trees, AST for short) by an

```
if test [ '-h'; '/etc/rancid/lg.conf' ] then
  rm [ '/etc/rancid/lg.conf' ]
fi
if test [ '-e'; '/etc/rancid/apache.conf' ] then
  rm [ '/etc/rancid/apache.conf' ]
fi
```

**Fig. 5.** `preinst` script of the `rancid-cgi` package in CoLiS

algebraic datatype in Why3. Then CoLiS semantics is defined by a set of inductive predicates [6] that encodes a chiefly standard, big-step operational semantics. The semantic rules cover the contents of variables and input/output buffers used during the evaluation of a CoLiS script, but they do not specify the contents of the file system and the behaviour of Posix commands. The judgements and rules are parameterised by bounds on the number of loop iterations and the number of (recursively) nested function calls to allow for formalising the correctness of the symbolic interpreter. The bounds are either a non-negative integer, or $\infty$ for unbounded execution, and keep constant throughout the evaluation of a CoLiS instruction. We refer to [6] for the details.

A concrete interpreter for the CoLiS language is implemented in Why3. Its formal specifications (preconditions and post-conditions) state the soundness of the interpreter, i.e., that any result corresponds to the formal semantics with unbounded number of loop iterations and unbounded nested function calls. The specifications are checked using automated theorem provers [23].

*Translation from shell to CoLiS.* This is done automatically, but it is not formally proven. Indeed, a formal semantics of shell was missing until very recently [21]. For the control flow constructs, the AST of the shell script is translated into the AST of CoLiS. For the strings (words in shell), the translation generates either a string CoLiS expression or a list of CoLiS expressions depending on the content of the shell string. This translation makes explicit the string evaluation in shell, in particular the implicit string splitting. At the present time, the translator rejects 23% of shell scripts because it does not cover the full constructs of the shell, e.g., usage of globs, variables with parameters, and advanced uses of redirections.

The conformance of the CoLiS script with the original shell script is not proven formally but tested by manual review and some automatic tests. For the latter, we developed a tool that automatically compares the results of the CoLiS interpreter on the CoLiS script with the results of the Debian default shell (`dash`) on the original shell script. This tool uses a test suite of shell scripts built to cover the whole constructs of the CoLiS language. The test suite allowed us to fix the translator and the formal semantics of CoLiS and, as an additional outcome, it revealed a lack of conformance between the Debian default shell and Posix[5].

[5] https://www.mail-archive.com/dash@vger.kernel.org/msg01683.html

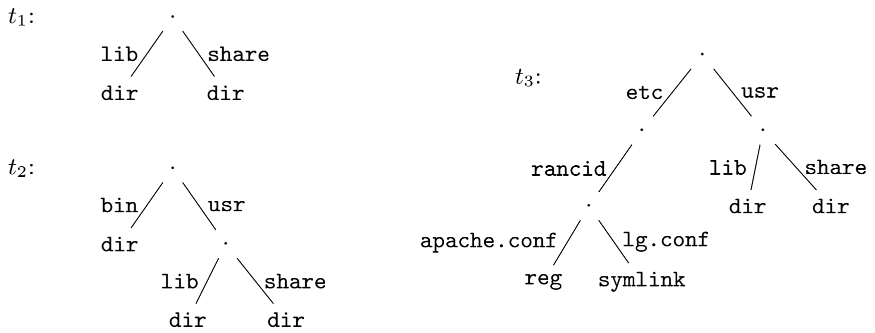

**Fig. 6.** Examples of feature trees showing directories ($t_1$), sub-directories ($t_2$), a regular file and a symbolic link ($t_3$).

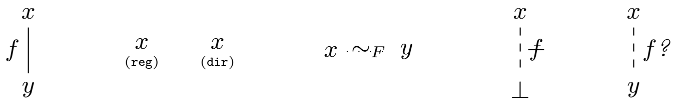

**Fig. 7.** Basic constraints, from left to right: a feature, a regular file node, a directory node, a tree similarity, a feature *absence*, a *maybe*

### 3.2 Feature trees and constraints

We employ models and logics to describe transformations of UNIX file systems. Feature trees [32,3,33] turn out to be suitable models for this case study. We have proposed a logic suitable to express file system transformations by extending previously existing logics. For the sake of space, we provide a concise overview of the model and logic used in this case study.

*Feature trees.* The models we consider here are trees with *features* (taken from $\mathcal{F}$, an infinite set of legal file names) on the edges, the `dir` *kind* on the nodes and any *kind* (`dir`, `reg` or `symlink`) on the leaves. Examples are given in Fig. 6.

*Constraints.* To specify properties of feature tree models, we modify our first order logic [26] to suit this case study's needs. For the sake of presentation, we use a graphical representation of quantifier-free conjunctive clauses of this logic. See the technical report [24] for a detailed presentation.

The core basic constraints are presented in Fig. 7. The *feature* constraint expresses that $y$ is a subtree of $x$ accessible from the root of $x$ *via* feature $f$. The *kind* constraints express that the root of a tree has the given kind (`dir`, `reg` or `symlink`). The *similarity* constraint expresses that $x$ and $y$ have the same children with the same names except for the children whose names are in $F$, a finite set of features, where they may differ.

For performance reasons, we added two more constraints; these do not increase the expressive power but help to prevent combinatorial explosion of formulas. The *absence* constraint expresses that either $x$ is not a directory or $x$ does not have a feature $f$ at its root. The *maybe* constraint expresses that either $x$ is not a directory, or it does not have a feature $f$ at its root, or it has one that leads to $y$.

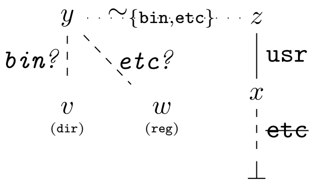

**Fig. 8.** A conjunctive clause

A model of a formula is a valuation that maps variables to feature trees. For instance, consider the valuation that associates $t_1$ to $x$, $t_2$ to $y$ and $t_3$ to $z$, where $t_1$, $t_2$ and $t_3$ are the trees defined in Fig. 6; it satisfies the formula in Fig. 8

*Satisfiability.* We designed a set of transformation rules [26] that turns any $\Sigma_1$-formula into an *irreducible form* that is either *false* or a satisfiable formula. This is convenient in our setting because we can detect unsatisfiable formulas as soon as possible and keep the irreducible form instead of the original formula, speeding up further computations. Our toolchain includes an implementation of this system, using an efficient representation of irreducible $\Sigma_1$-formulas as trees themselves. Finally, the system of rules is also extended to a quantifier elimination procedure, showing that the whole first-order logic is decidable.

### 3.3 Specifications of UNIX commands

The specification of the UNIX commands uses our feature tree logic to express their effect on the file system. The specification formalises the description given in natural language in the POSIX standard [22, Chapter Utilities] and, for some commands, in GNU manual pages. We only specified (most of) the UNIX commands called by the maintainer scripts.

The full specification is available in a separate technical report [24]. We present here its main ingredients. A UNIX command has the form: "`cmd options paths`", where "`cmd`" is a command name, "`options`" is a list of options, and "`paths`" is one or more absolute or relative paths (i.e., sequence of file names and symbols "`.`" and "`..`"). For each combination of command name and option, we provide a list of formulas specifying the success and failure cases. A success or failure case formula has two free variables $r$ and $r'$, which represent the root of the file system before and after the command execution. For some combinations of command names and options, the specification is not provided, but computed by the symbolic execution of a CoLiS script. This script captures the command behaviour by calling other (primitive) commands.

*Path resolution.* An important ingredient in command specification is the constraint encoding the resolution of a path in the file system. For this, we define a predicate $\texttt{resolve}(r, cwd, p, z)$ stating that "when the root of the file system is $r$ and the current working directory is the sequence of features $cwd$, the path $p$ resolves and goes to variable $z$". The constraint defining this predicate is a $\Sigma_1$

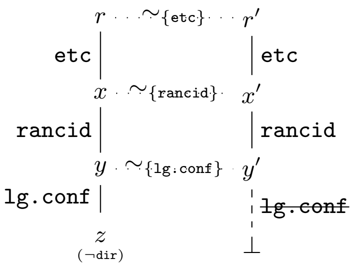

**Fig. 9.** Specification of success case for `rm /etc/rancid/lg.conf`

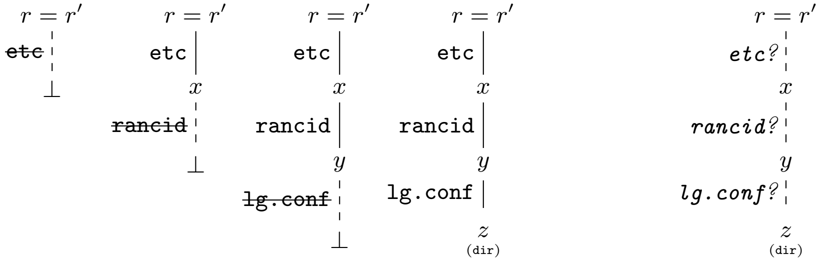

**Fig. 10.** Specification of error cases of `rm /etc/rancid/lg.conf`: explicit cases on the left, compact specification on the right conjunction of basic constraints; it does not deal with symbolic link files on the path. For example, the constraint `resolve`$(r, cwd,$ `/etc/rancid/lg.conf`$, z)$ is represented by the path starting from $r$ and ending in $z$ in Fig. 9.

For some commands, a failure of path resolution may cause the failure of the command. To specify these failure cases, we have to use the negation of the predicate `resolve`, which generates a number of clauses which is linear in the length of the resolved path. Fig. 10 shows, in the three left-most constraints, the error cases for the resolution of the path to `/etc/rancid/lg.conf`. Because the internal representation of formulas keeps only conjunctive clauses, this may produce a state explosion of constraints when the command uses several paths. To obtain a compact internal representation of these error cases, we employ the *maybe* shorthand, as shown on the right of Fig. 10.

Let us consider the command `rm /etc/rancid/lg.conf`. Its specification includes one success case, given on Fig. 9: the resolution of the path `/etc/rancid/lg.conf` succeeded in the initial file system denoted by $r$, and the resulting file system, denoted by $r'$ is similar to $r$ except for the absence of the feature `lg.conf`. The specification also includes one error case given on Fig. 10, where the path cannot be resolved to a regular path, and therefore the initial and final file systems are the same.

It is important to notice that specifications of commands are *parameterised* by their path(s) argument(s): for each concrete value of such paths, an appropriate constraint is produced. This fact is essential for using our symbolic engine, because the variables of a constraint denote nodes of the file system, but there is no notion of variable denoting file names or paths.

### 3.4 Analysis by symbolic execution

With a similar approach as for the concrete interpreter (Section 3.1), we designed and implemented a symbolic interpreter for the CoLiS language in Why3. Guided by a proof-of-concept symbolic interpreter for a simple IMP language [5], the main design choices for the symbolic interpreter for CoLiS are:

- Variables are *not* interpreted abstractly: when executing an installation script, the concrete values of the variables are known. On the other hand, the state of the file system is not known precisely, and it is represented symbolically using a feature tree constraint.
- The symbolic engine is generic with respect to the utilities: their specifications in terms of symbolic input/output relations are taken as parameters.
- The number of loop iterations and the number of (recursively) nested function calls [6]) is bounded a priori, the bound is given by a global parameter set at the interpreter call.

The Why3 code for the symbolic interpreter is annotated with post-conditions to express that it computes an *over-approximation* [5] of the concrete states that are reachable without exceeding the given bound on loop iterations. This property is formally proven using automated provers. The OCaml code is automatically extracted from Why3, and provides an executable symbolic interpreter with strong guarantees of soundness with respect to the concrete formal semantics.

Notice that our symbolic engine neither supports parallel executions, nor file permissions or file timestamps. This is another source of over-approximation, but also under-approximation, meaning that our approach can miss bugs whose triggering relies on the former features.

The symbolic interpreter provides a symbolic semantics for the given script: given an initial symbolic state that represents the possible initial shape of the file system, it returns a triple of sets of symbolic input/output relations, respectively for normal result, error result (corresponding to non-zero exit code) and result when a loop limit is reached. Error results are unexpected for Debian maintainer scripts, and these cases have to be inspected manually. To help this inspection, a visualisation of symbolic relations was designed, as already described in Fig. 4.

### 3.5 Scenarios

So far, we have presented how we analyse individual maintainer scripts. In reality, the Debian policy specifies in natural language in which order and with which arguments these scripts are invoked during package installation, upgrade, or removal (see, for instance, Fig. 2). We have specified these scenarios in a loop-free custom language. These scenarios define what happens after the success or the failure of a script execution. They also specify when the static content is unpacked. Furthermore, our toolchain allows to define the assumptions that can be made on an initial filesystem before executing a scenario, for instance the File System Hierarchy Standard [38]. Our toolchain reports on packages that may remain in an unexpected state after the execution of one of these scenarios.

For instance, the installation scenario of the package *rancid-cgi* may leave that package in the state *not-installed*, which is reported by our toolchain using the diagram in Fig. 4.

## 4 Results and impact

### 4.1 Coverage of the case study

The tools used and the datasets analysed during the current study are available in the Zenodo repository [36].

We execute the analysis on a machine equipped with 40 hyperthreaded Intel Xeon CPU @ 2.20GHz, and 750GB of RAM. To obtain a reasonable execution time, we limit the processing of one script to 60 seconds and 8GB of RAM. The time limit might seem low, but the experience shows that the few scripts (in 30 packages) that exceed this limit actually require hours of processing because they make a heavy use of `dpkg-maintscript-helper`. On our corpus of 12 592 packages with 28 814 scripts, the analysis runs in about half an hour.

All of those scripts that are syntactically correct with respect to the Posix standard (99.9%) are parsed successfully by our parser. The translation of the parsed scripts into our intermediary language CoLiS succeeds for 77% of them; the translation fails mainly because of the use of globs, variables with parameters and advanced uses of redirections.

Our toolchain then attempts to run 113 328 scenarios (12 592 packages with scripts, 9 scenarios per package). Out of those, 45 456 scenarios (40%) are run completely and 13 149 (12%) partially. This is because scenarios have several branches and although a branch might encounter failure, we try to get some information on execution of other branches. For the same reason, one scenario might encounter several failures. In total, we encounter 67 873 failures. The origins of failures are multiple, but the two main ones are (i) trying to execute a scenario that includes a script that we cannot convert (28% of failures), or (ii) the scripts might use commands unsupported by our tools, or unsupported features of supported commands (71% of failures).

Among the scenarios that we manage to execute at least partially, 19 reach an unexpected end state. These are potential bugs. We have examined them manually to remove false positives due to approximations done by our methodology or the toolchain. We discuss in Section 4.3 the main classes of true bugs revealed by this process.

### 4.2 Corpus mining

The latest version of the Debian *sid* distribution on which we ran our tools dates from October 6, 2019. It contains 60 000 packages, 12 592 of which contain at

**Table 1.** Bugs found between 2016 and 2019 in Debian *sid* distributions

| *Bugs* | *Closed* | *Detected by* | *Reports* | *Examples* |
|---|---|---|---|---|
| 95 | 56 | parser | [9] | not using `-e` mode |
| 6 | 4 | parser & manual | [15] | unsafe or non-Posix constructs |
| 34 | 24 | corpus mining | [8,10] | wrong options, mixed redirections |
| 9 | 7 | translation | [11] | wrong test expressions |
| 5 | 2 | symbolic execution | [13,17,15] | try to remove a directory with `rm` |
| 3 | 3 | formalisation | [12] | bug in `dpkg-maintscript-helper` |
| 152 | 96 | | | |

least one maintainer script, which leads to 28 814 scripts. In total, these scripts contain 442 364 source lines of code, 15 lines on average, and up to 1 138 for the largest script. Among them we find 220 `bash` scripts, 2 `dash` scripts, 14 `perl` scripts, and one ELF executable – the rest are Posix shell scripts.

In the process of designing our tools, and in order to validate our hypotheses, we ran statistical analysis on this corpus of scripts. The construction of our tool for statistical analysis is described in a technical report [25] where we also detail a few of our findings. To summarise, analysing the corpus revealed that:

- Most variables in scripts were used as constants: only 3 008 scripts contain variables whose value actually changes.
- There are no recursive functions in the whole corpus.
- There are 2 300 scripts that include a while loop. 93% of the while loops occur in a pipe reading the output of `dpkg -L` and are an idiosyncrasy that is proper to some shell languages. They can be translated to "foreach" loops in a properly typed language.
- The huge majority of redirections are used to hide the standard output or merge it into the error output.

This analysis had an important impact on the project by guiding the design choices of CoLiS, which Unix commands we should specify and in which order, etc. This also helped us to discover a few bugs, e.g., scripts invoking Unix commands with invalid options.

### 4.3 Bugs found

We ran our toolchain on several snapshots of the Debian *sid* distribution taken between 2016 and 2019, the latest one being October 6, 2019. We reported over this period a total of 152 bugs to the Debian Bug Tracking System [37]. Some of them have immediately been confirmed by the package maintainer (for instance, [16]), and 96 of them have already been resolved.

Table 1 summarises the main categories of bugs we reported. Simple lexical analysis already detects 95 violations of the Debian Policy, for instance scripts that do not specify the interpreter to be used, or that do not use the `-e` mode [9]. The shell parser (Section 3.1) detects 3 scripts that use shell constructs not allowed by the Posix standard, or in a context where the Posix standard states that the behaviour is undefined [15]. There are also 3 miscellaneous bugs, like using unsafe shell constructs. The mining tool (Section 4.2) detects 5 scripts that invoke Unix commands with wrong options and 29 scripts that mix up redirection of standard-output and standard-error. The translation from the shell to the CoLiS language (Section 3.1) detects 9 scripts with wrong test expressions [11]. These may stay unnoticed during superficial testing since the shell confuses, when evaluating the condition of an if-then-else, an error exception with the Boolean value *False*. Inspection of the symbolic semantics extracted by the symbolic execution (Section 3.4) finds 5 scripts with semantic errors. Among these is the bug [16] of the package *rancid-cgi* already explained in Section 2.4. During the formalisation of Debian tools (see Section 3.3), we found 3 bugs. These include in particular a bug [12] in the `dpkg-maintscript-helper` command which is used 10 306 times in our corpus of maintainer scripts, and was fixed in the meantime.

### 4.4 Lessons learnt

One basic problem when trying to analyse maintainer scripts is to understand precisely the meaning of the policy document. For instance, one of the more intriguing requirements is that maintainer scripts have to be idempotent (Section 6.2 in [4]). While it is common knowledge that a mathematical function $f$ is idempotent when $f(f(x)) = f(x)$ for any $x$, the meaning is much less clear in the context of Debian maintainer scripts as the policy goes on to explain "If the first call failed, or aborted half way through for some reason, the second call should merely do the things that were left undone the first time, if any, and exit with a success status if everything is OK." We suppose that this refers to causes of error external to the script itself (power failure, full disk, etc.), and that there might be an intervention by the system administrator between the two invocations. Since we cannot even explain in natural language what precisely that means, let alone formalise it, we decided to model at the moment only a rough under-approximation of that property that only compares executions by their exit code. This allowed us to detect a bug [14].

We found that identifying bugs in maintainer scripts always requires human examination. Automated tools allow to point out potential problems in a large corpus, but deciding whether such a problem actually deserves a bug report, and of what severity level, requires some experience with the Debian processes. This is most visible with semantic bugs in scripts, since an error exit code does not imply that there is a bug. Indeed, if a script detects a situation it cannot handle then it *must* signal an error and produce a useful error message. Deciding whether a detected error case is justified or accidental requires human judgement.

Filing bug reports demands some caution, and observance of rules and common practices in the community. For instance, the Debian Developers Reference [18] requires approval by the community before so-called *mass bug filing*. Consequently, we always sought for advice before sending batches of bugs, either on the Debian developers mailing list, or during Debian conferences.

## 5 Conclusion

The corpus of Debian maintainer scripts is an interesting case study for analysis due to its size, the challenging features of the scripting language, and the relational properties it requires to analyse. The results are very promising. First, we reported 152 bugs [37] to the Debian Bug Tracking system, 96 of which have already been resolved by Debian maintainers. Second, the toolchain performs the analysis of a package in seconds and of the full distribution in less than a hour, which makes it fit for integration in the workflow of Debian maintainers or for quality assurance at the the level of the whole distribution. Integration of our toolchain in the `lintian` tool will not be possible since it would add a lot of external dependencies to that tool, and since the reports generated by our tool still require human evaluation (see Section 4.4).

This study had several additional outcomes. The toolchain includes tools for parsing and light static analysis of shell scripts [30], an engine for the symbolic execution of imperative languages based on first-order logics representation of program configurations [5], and an efficient decision procedure for feature tree logics. We also provide a formal specification of Posix commands used in Debian scripts in terms of a first-order logic [24].

We are not aware of a project dealing with this kind of problem or obtaining comparable results. To our knowledge, the only existing attempt to analyse a complete corpus of package maintainer scripts was done in the context of the Mancoosi project [19]. In this work, the analysis, mainly syntactic, resulted in a set of building blocks used in maintainer scripts that may be used in a DSL. In a series of papers [20,28,29], Ntzik et al. consider the formal reasoning on the Posix scripts manipulating the file system based on (concurrent) separation logic. Not only do they employ a different logic (a second-order logic), but they also focus on (manual) proof techniques for correctness and not on automatic techniques for finding bugs. Moreover, they consider general scripts and properties that are not relational (like idempotency). There have been few attempts to formalise the shell. Greenberg [21] recently offers an executable formal semantics of Posix shell that will serve as a foundation for shell analysis tools. Abash [27] contains a formalisation of parts of the bash language and an abstract interpretation tool for the analysis of arguments passed by scripts to Unix commands; this work focused on identifying security vulnerabilities.

The successful outcome of this case study revealed new challenges that we aim to address in future work. In order to increase the coverage of our analysis and the acceptance by Debian maintainers, the translation from shell should cover more features, additional Unix commands should be formally specified, and the model should capture more features of the file system, e.g., permissions, or symbolic links. The efficiency of the analysis can still be improved by using a more compact representation of disjunctive constraints in feature tree logics or by exploiting the genericity of the symbolic execution engine to include other logic based symbolic representations that may be more efficient and precise. Finally, we want to use the computed constraints on scenarios to check new properties of scripts like equivalence of behaviours.
